# Supplementary material for: South Arabia’s prehistoric monument landscape shows social resilience to climate change
Source: PLoS One. 2025 May 28;20(5):e0323544. doi: 10.1371/journal.pone.0323544 (PMC12118824; doi:10.1371/journal.pone.0323544)
Supplement: S1 Fig — (PDF) [file pone.0323544.s004.pdf]

S4-Fig

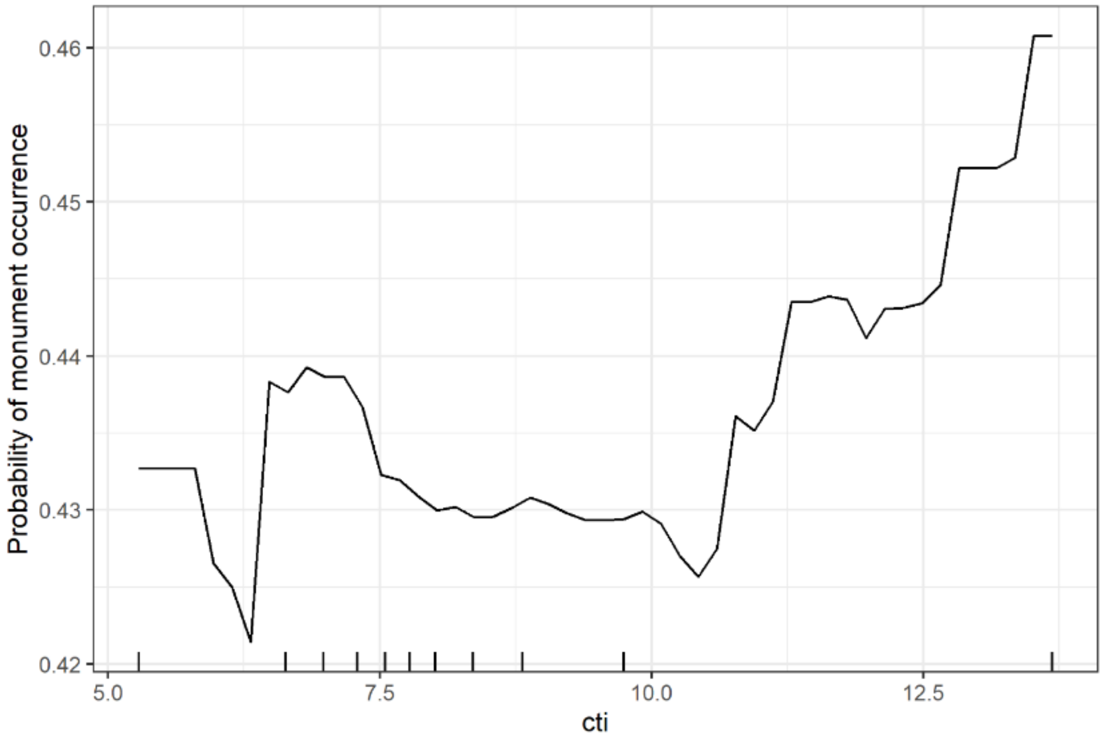

Compound Topographic Index

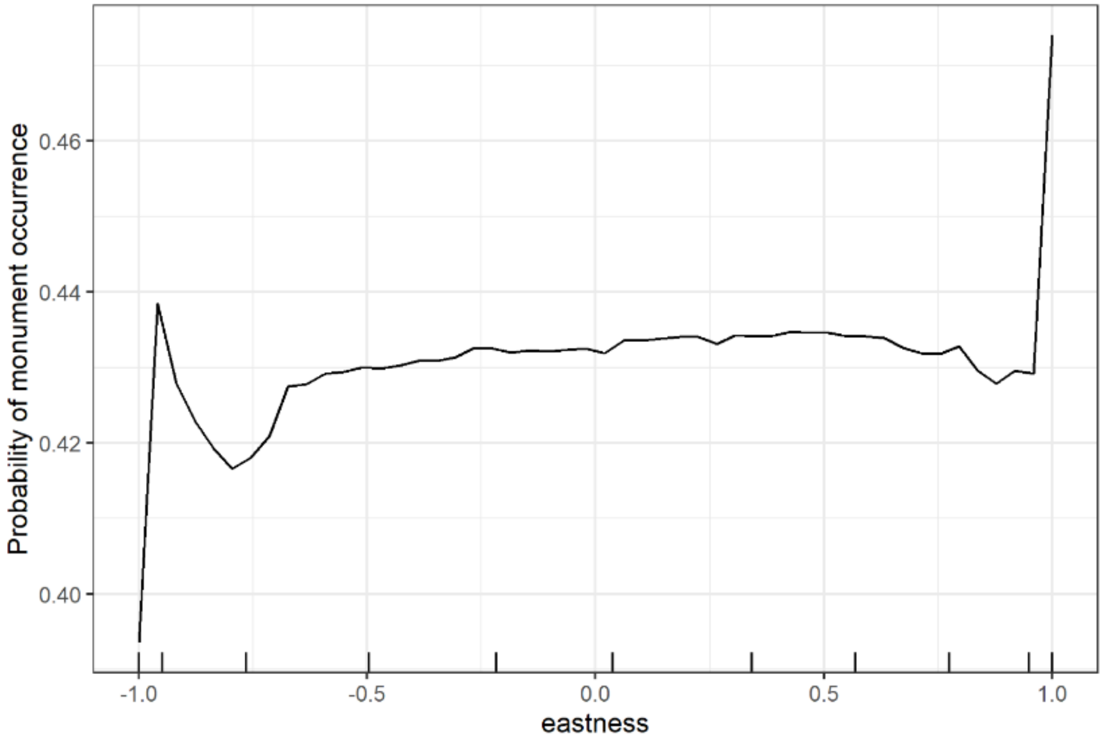

Eastness

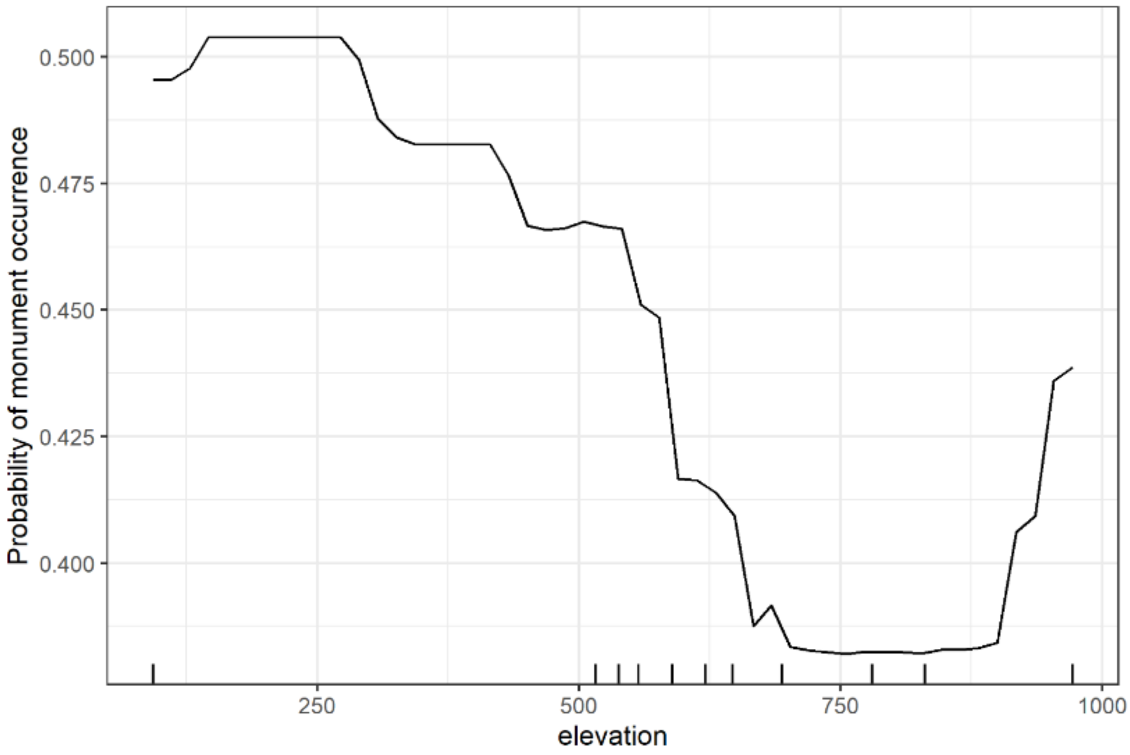

Elevation

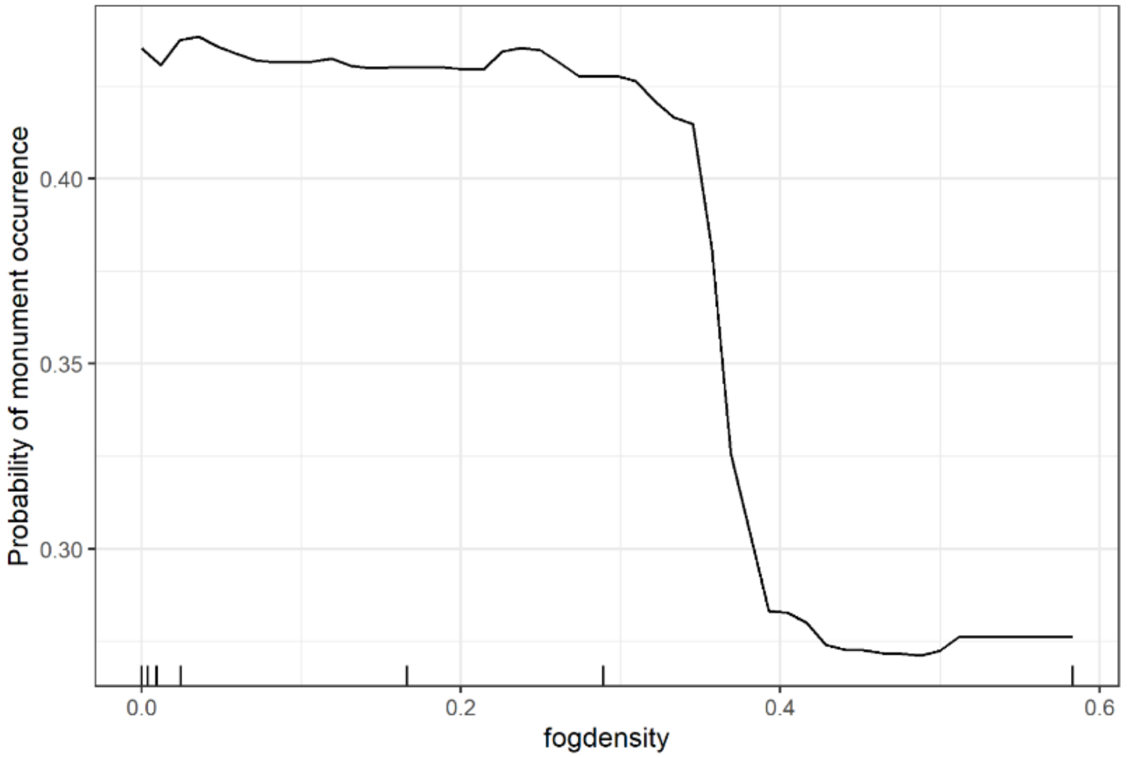

Fog Density

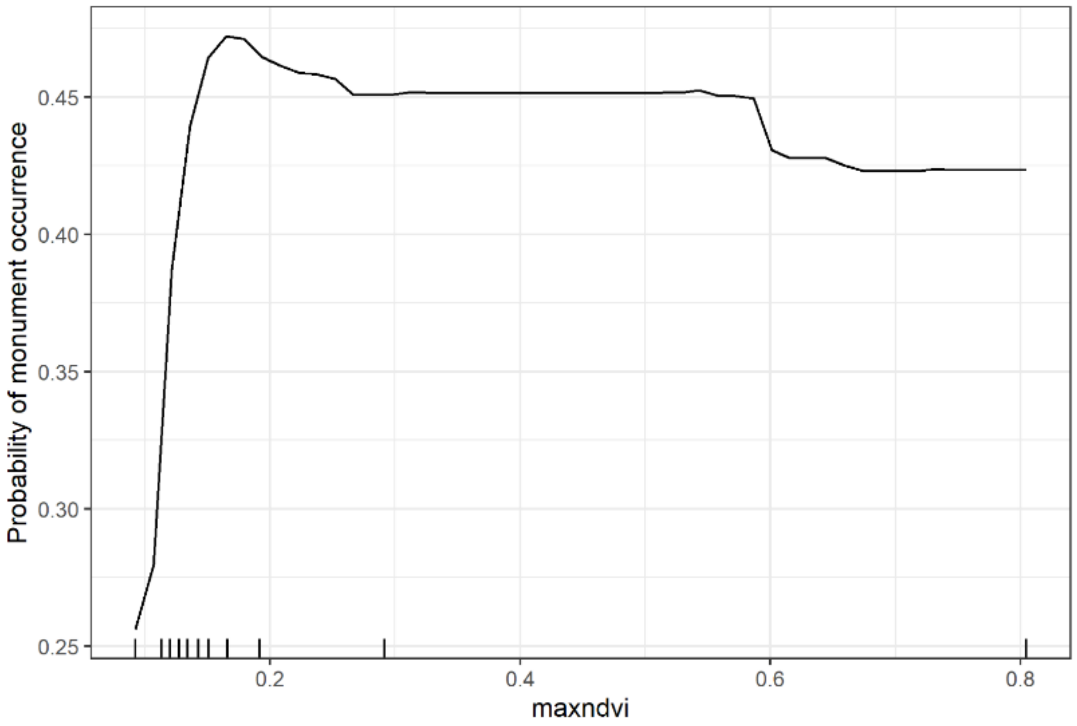

Maximum NDVI

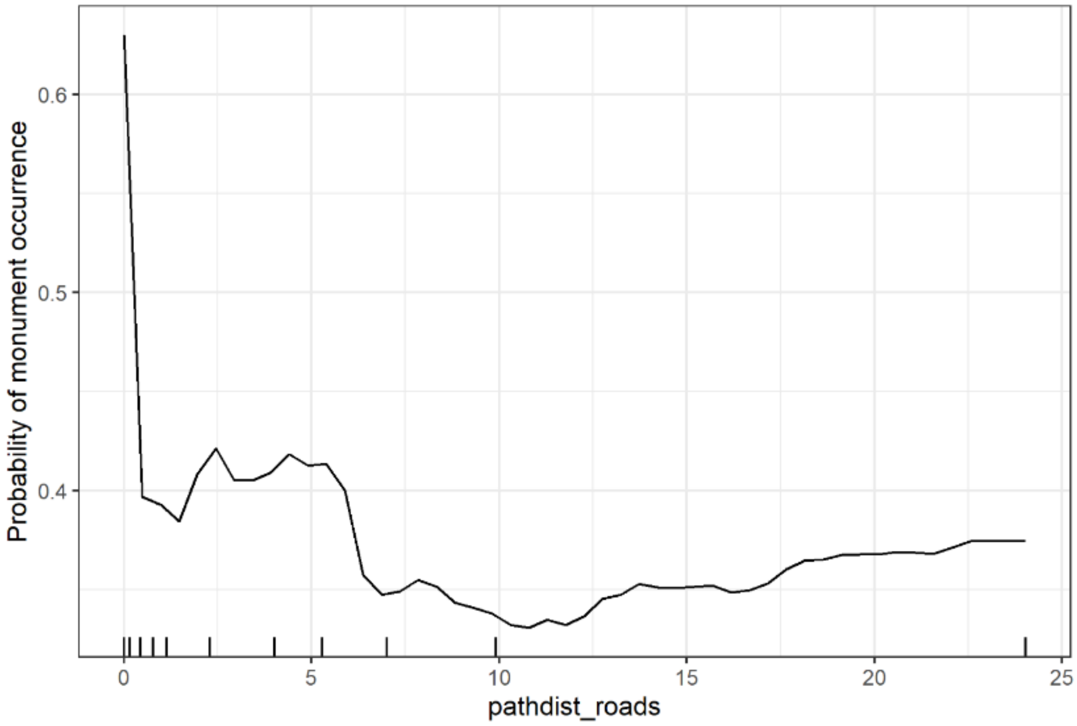

Path Distance to Roads

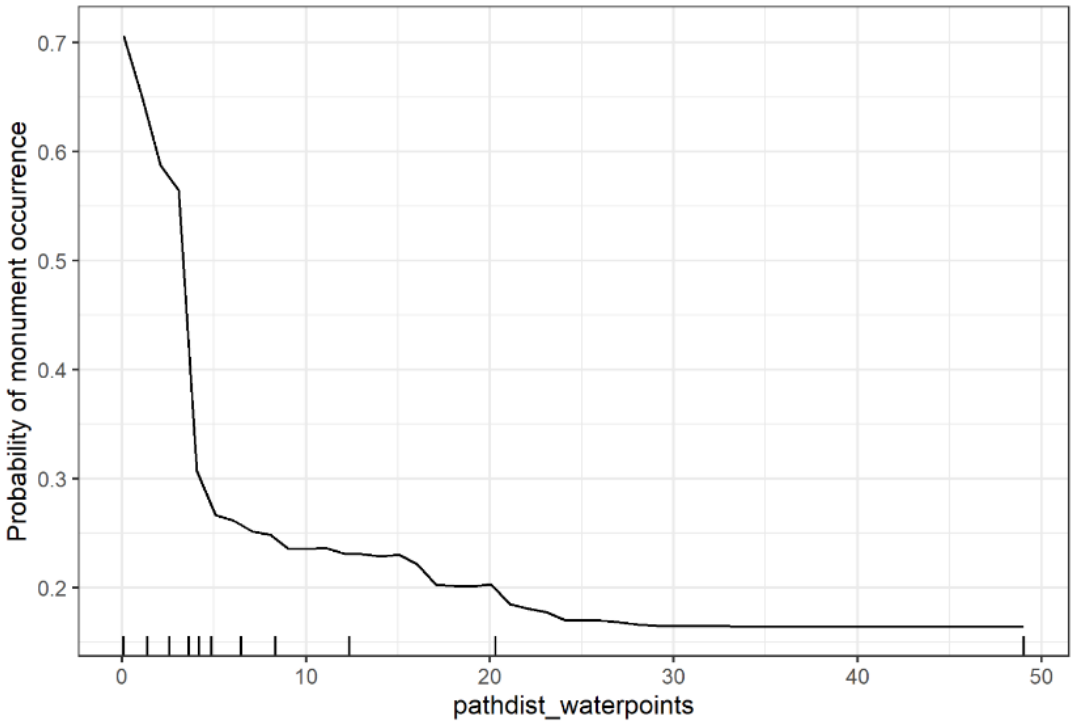

Path Distance to Water Points

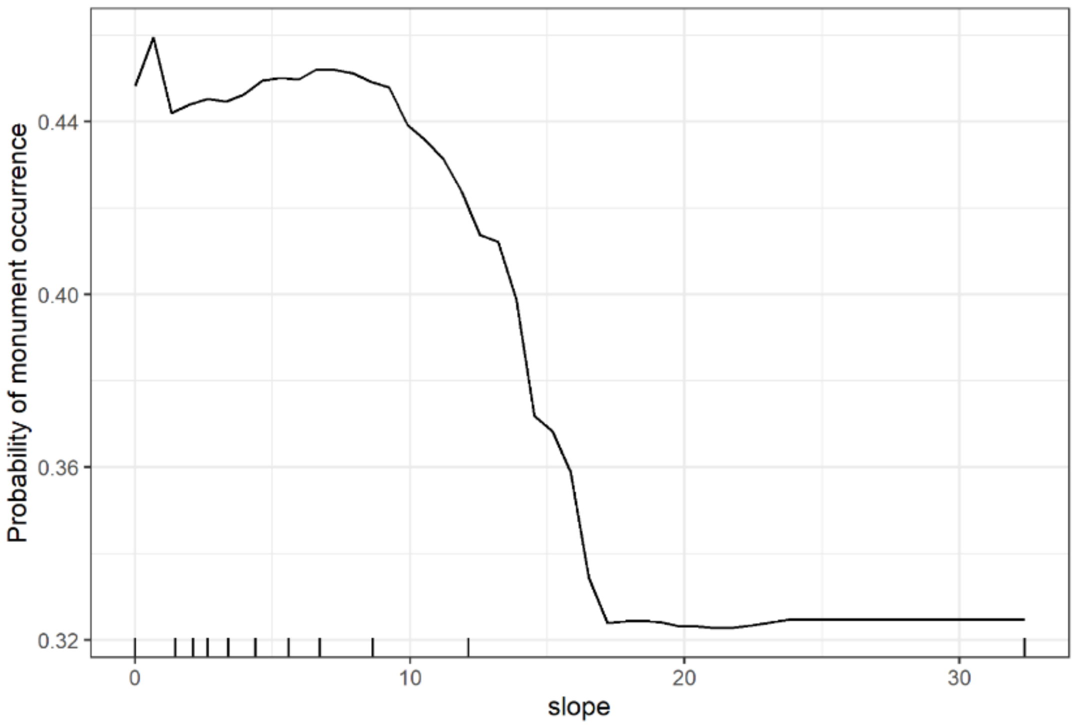

Slope

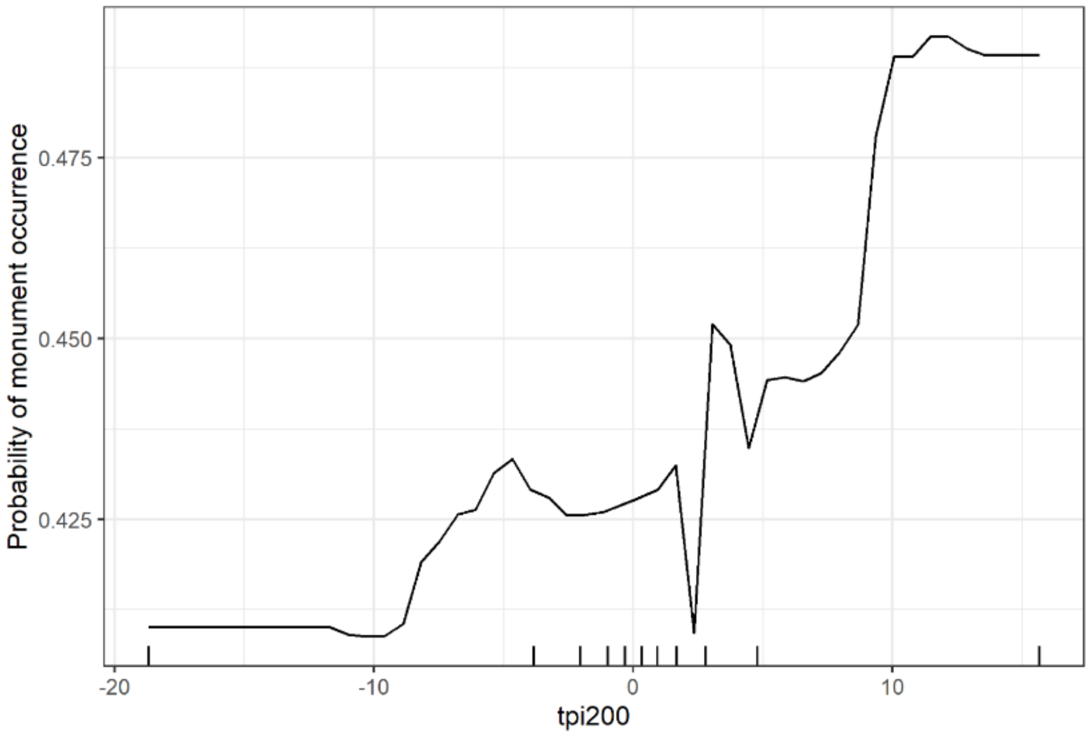

Topographic Position Index (r=200)

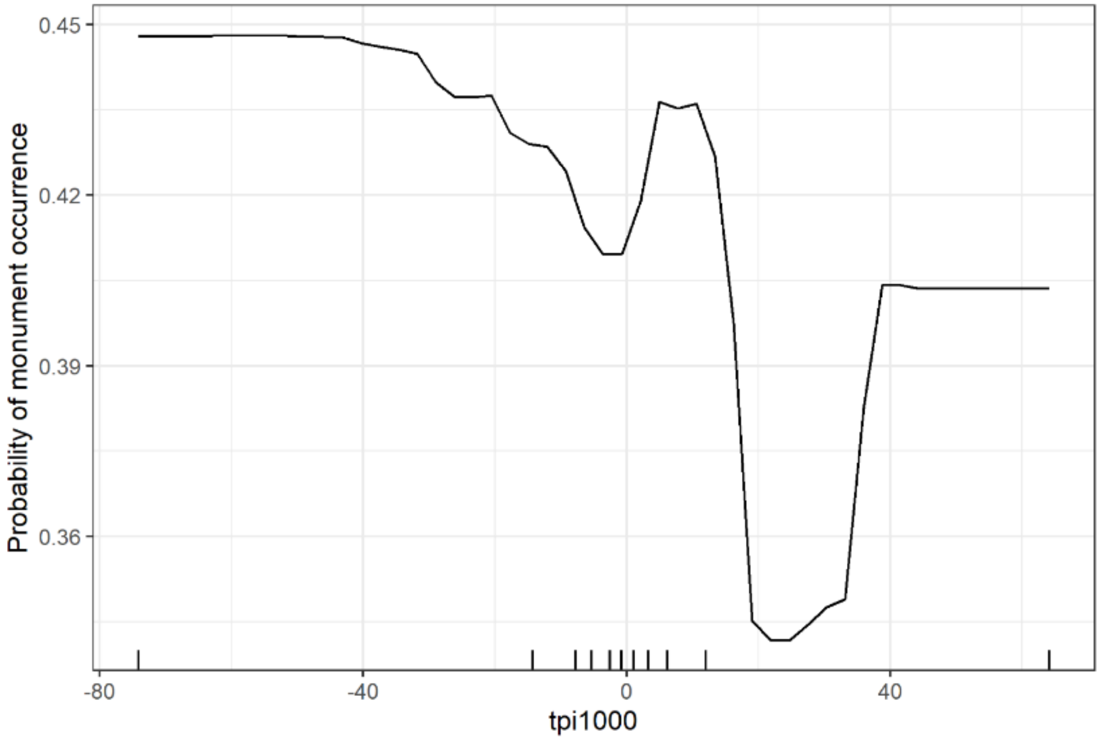

Topographic Position Index (r=1000)

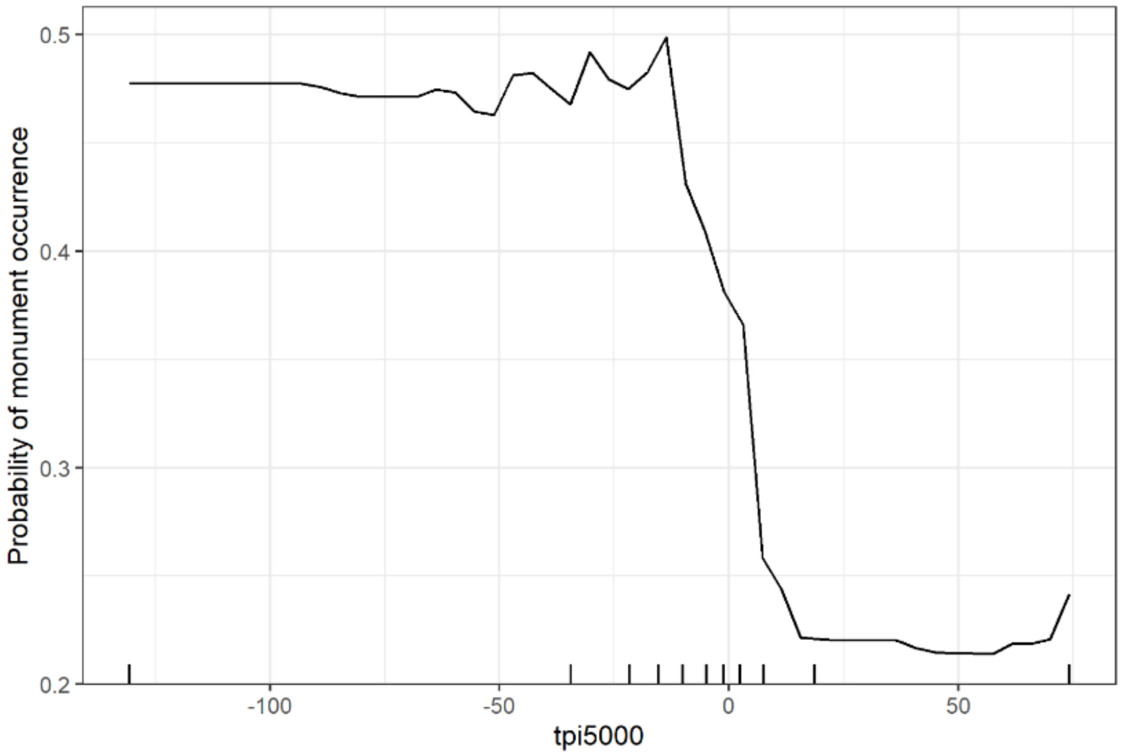

Topographic Position Index (r=5000)

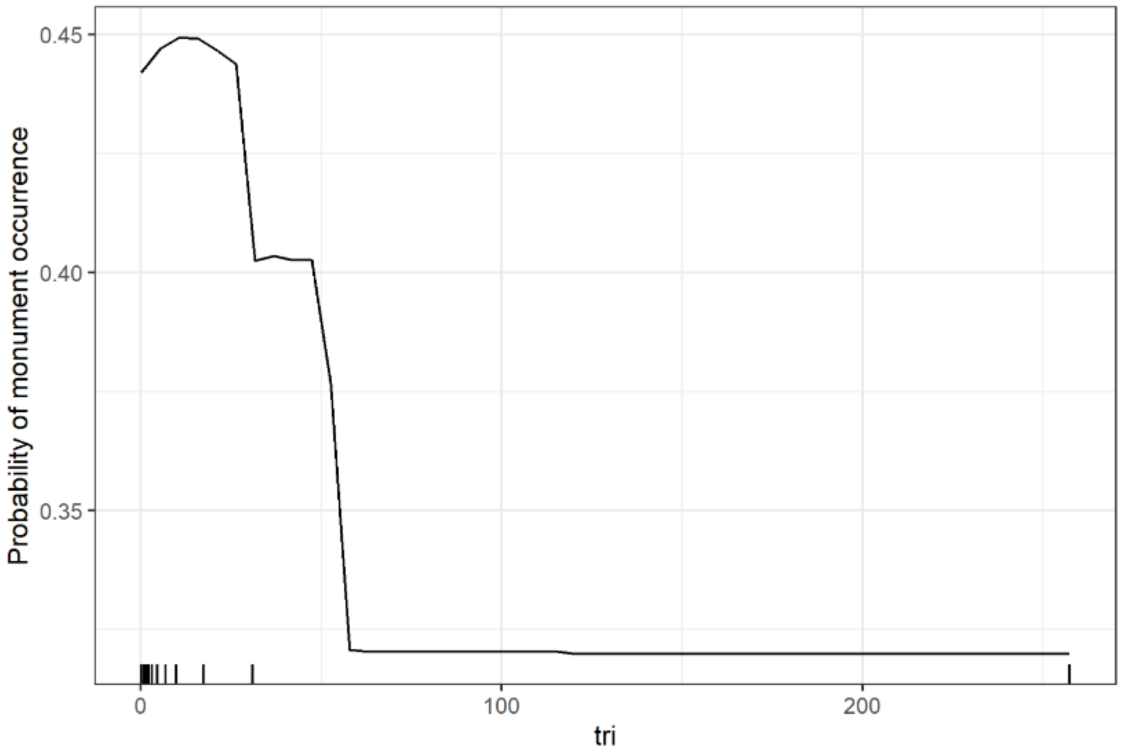

Terrain Ruggedness Index
