# Supplementary figures and images for: South Arabia’s prehistoric monument landscape shows social resilience to climate change
Source: PLoS One. 2025 May 28;20(5):e0323544. doi: 10.1371/journal.pone.0323544 (PMC12118824; doi:10.1371/journal.pone.0323544)

**S5-Fig**

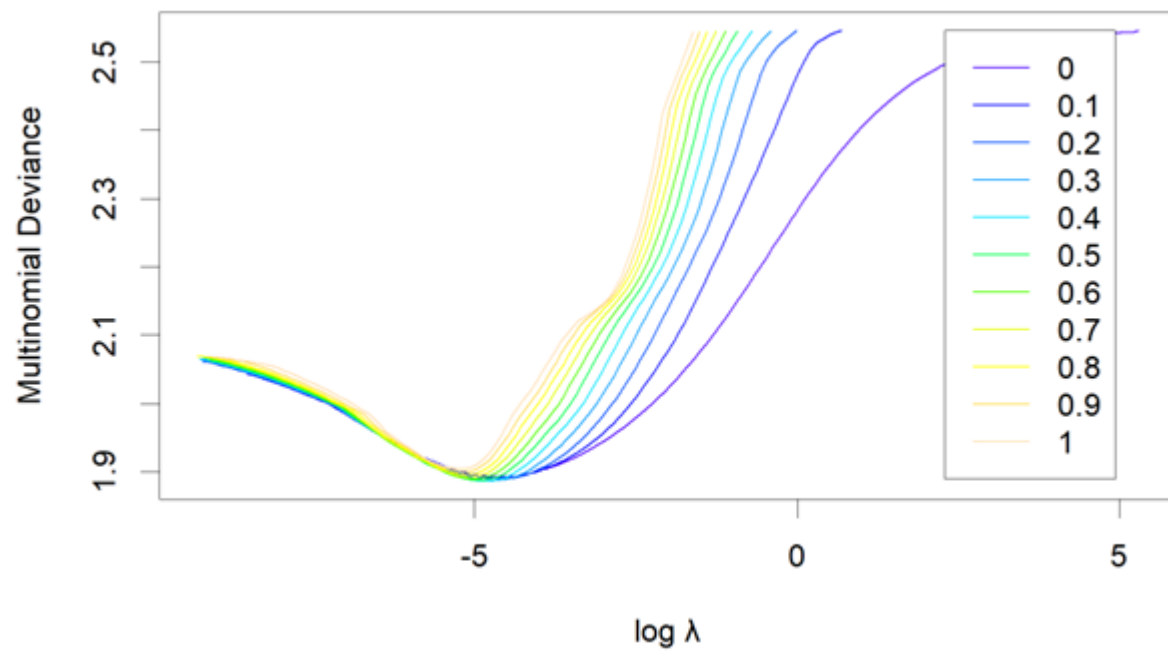

Supplement: S2 Fig — We used the model with α = 0.4 (elastic net regression), which had the lowest multinomial deviance. (PDF) [file pone.0323544.s005.pdf]

S6-Fig

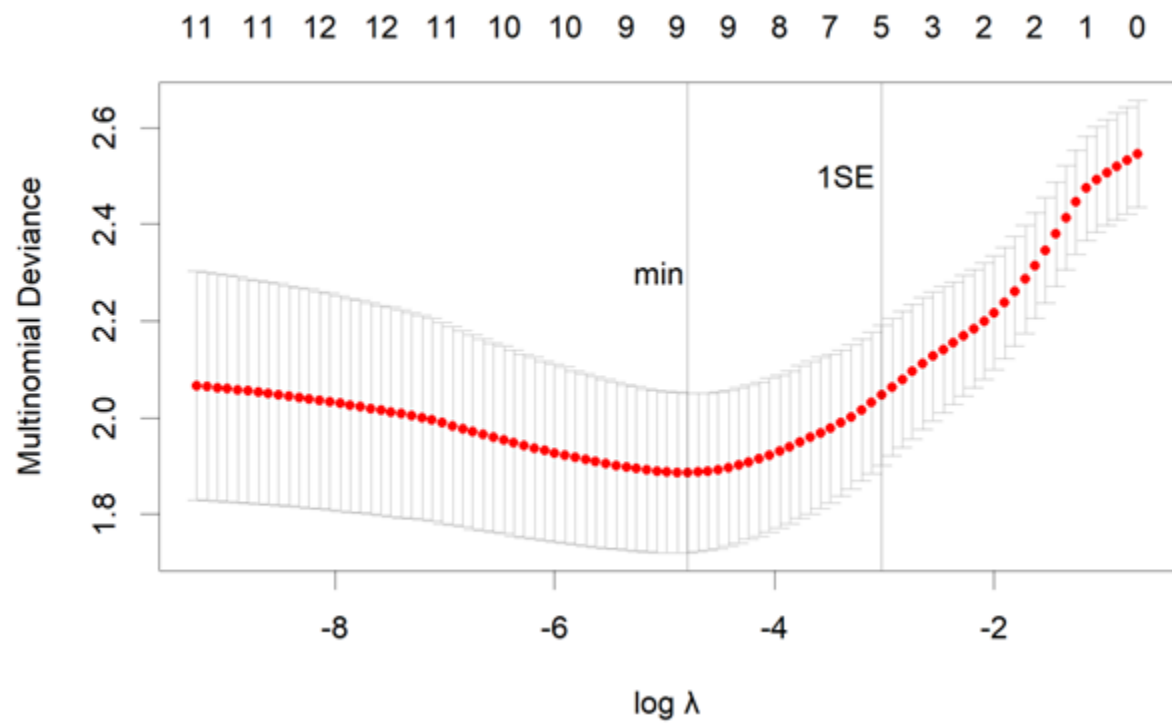

Supplement: S3 Fig — We used the ‘one-standard error’ rule, to select the model with λ = 0.049 (log λ = -3.023). (PDF) [file pone.0323544.s006.pdf]

**S7-Fig**

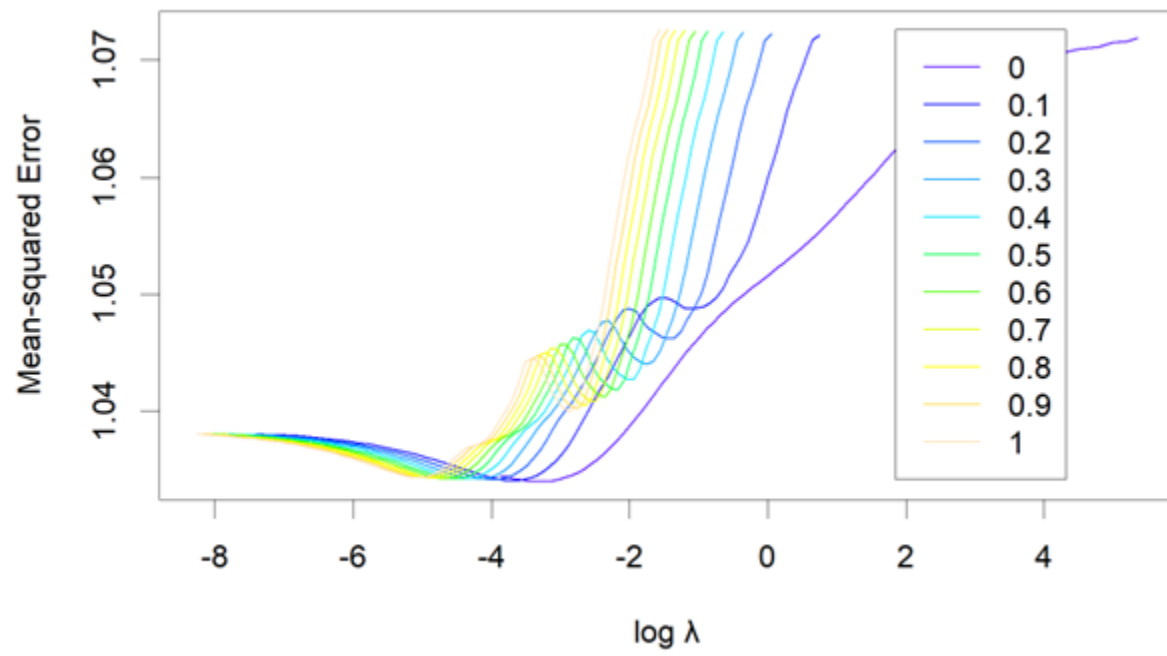

Supplement: S4 Fig — We used the model with α = 0.1 (elastic net regression), which had the lowest mean-squared error. (PDF) [file pone.0323544.s007.pdf]

**S8-Fig**

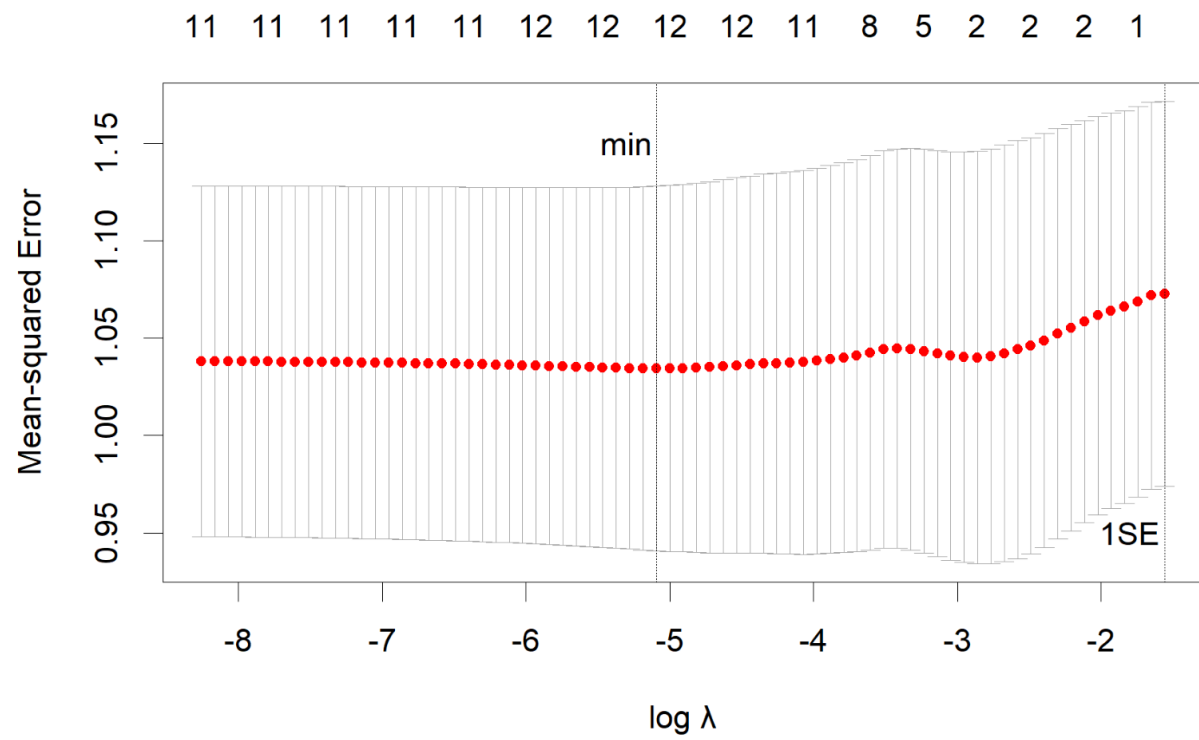

Supplement: S5 Fig — We selected the model with the minimum λ = 0.129 (log λ = -2.047). (PDF) [file pone.0323544.s008.pdf]

**S9-Fig**

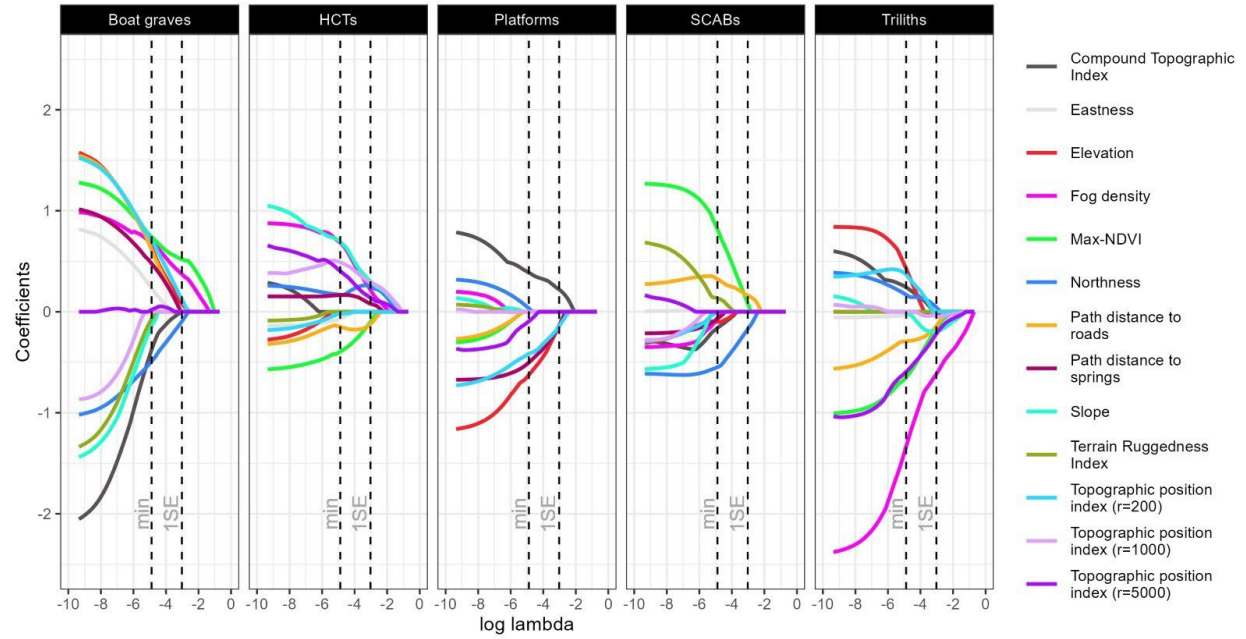

Supplement: S6 Fig — As λ decreases, the maximum permissible value of the L1 norm increases and more coefficients enter the model. Variables enter the model based on their true linear regression coefficient, and therefore variables that enter the model early have a higher predictive power, compared to those that enter the model later. When a new variable enters the model, it affects the slope of the coefficient paths of the other predictors, depending on the magnitude of collinearity. (PDF) [file pone.0323544.s009.pdf]

**S10-Fig**

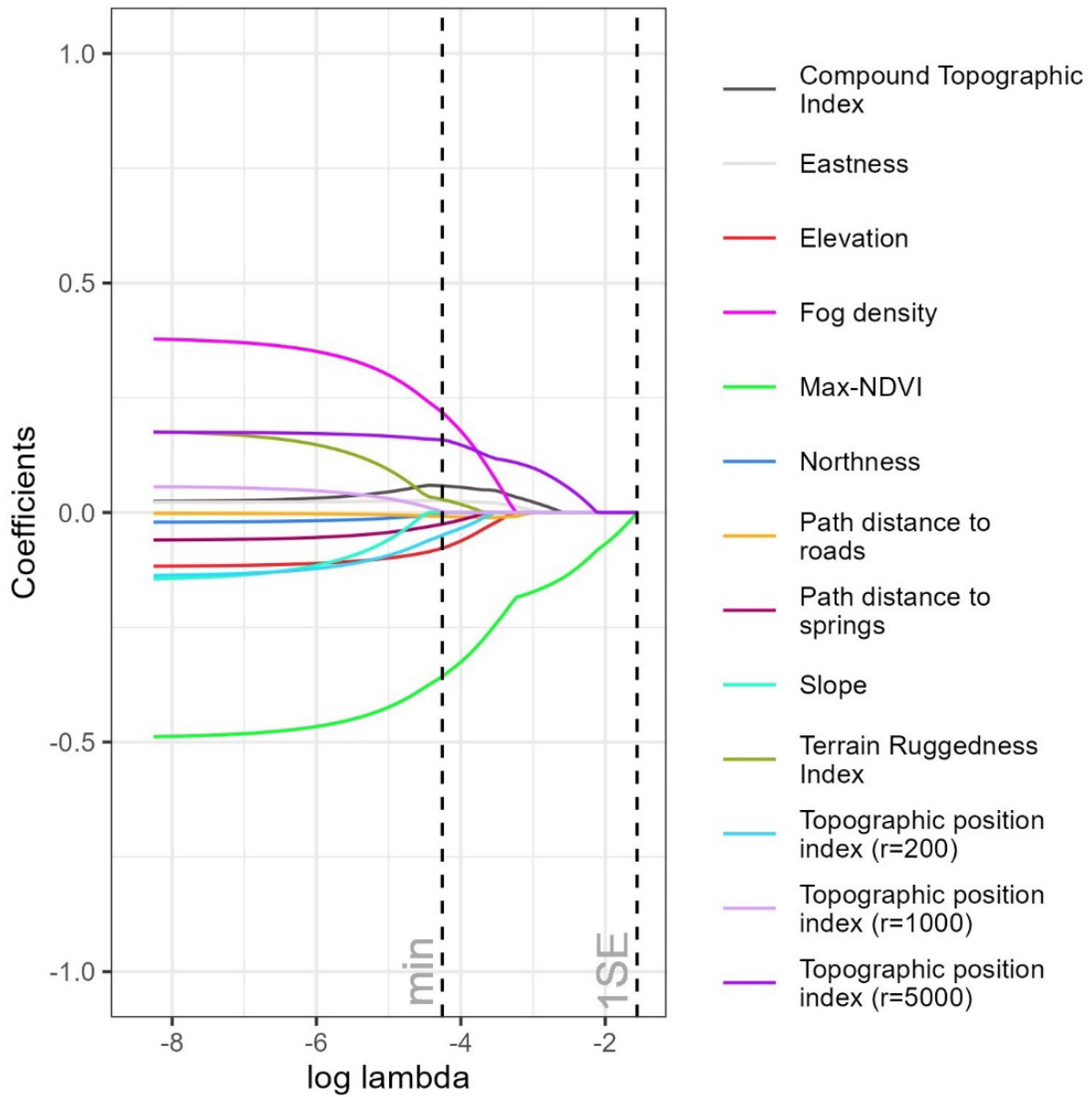

Supplement: S7 Fig — The regularized multiple linear regression assessed whether labor input responded to environmental factors; however, using the ‘one-standard error’ rule, no variables were preserved in the model. Eleven of the twelve environmental variables were preserved in the minimum lambda model. The variables with the highest coefficients entered the model early and have the greatest predictive power. Notably, labor input (total stone volume) increased with fog density and topographic position at large scales but decreased with maximum NDVI. Like the results of bootstrap aggregating, the minimum lambda result suggests a threshold in population dispersal at which humans engage monuments for messaging-while-absent. At the higher population densities supported in the vegetated mountains, people had sufficient direct contact that monuments were not needed as social touchstones. Fog density is high in the near Nejd backslope of the plateau, where mobile families would readily find seasonal grazing and yet be infrequently in contact, using monuments to communicate. (PDF) [file pone.0323544.s010.pdf]

**S11-Fig**

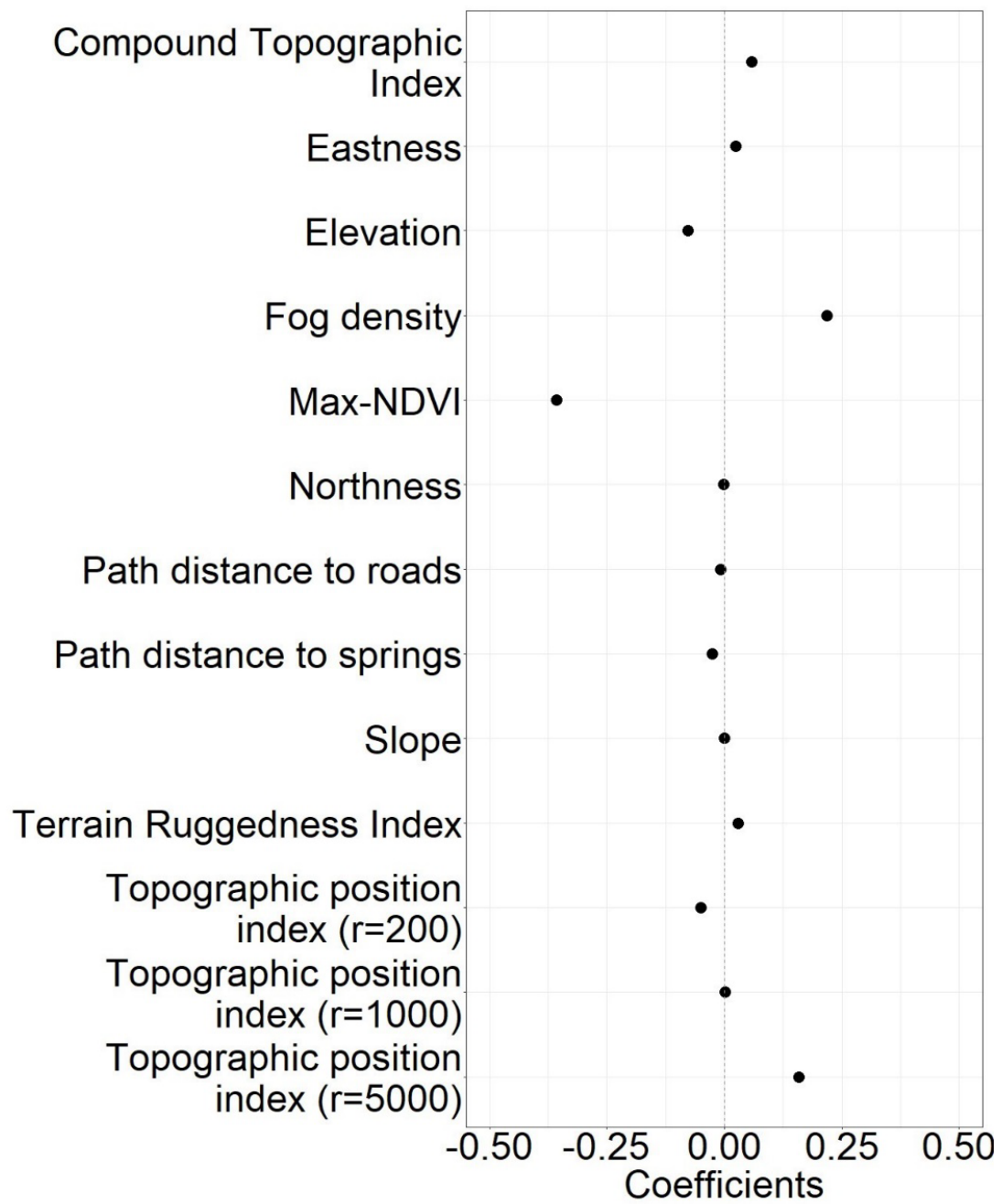

Supplement: S8 Fig — A positive coefficient indicates that stone volume increases with that variable, whilst a negative coefficient shows an inverse relationship between stone volume and that variable. We also analyzed whether labor input responded to environmental factors within the most common monument types; HCTs (n = 100) and Triliths (n = 138). No variables were preserved in the ‘one-standard error’ rule models, but in the minimum lambda models, total stone volumes for HCTs increased with fog density (coef = 0.0798), whilst for Triliths, total stone volumes decreased with increasing slope steepness (coef = -0.0677). These results are also consistent with bootstrap aggregating and with an interpretation that HCT (episodic) lay within a zone where labor could be convened, (closer to the vegetated mountains), while the accretive triliths decline in size where labor expenditures rise in carrying stones up and down steep inclines. (PDF) [file pone.0323544.s011.pdf]
