# Supplementary material for: South Arabia’s prehistoric monument landscape shows social resilience to climate change
Source: PLoS One. 2025 May 28;20(5):e0323544. doi: 10.1371/journal.pone.0323544 (PMC12118824; doi:10.1371/journal.pone.0323544)
Supplement: Attachment — Submitted filename: Response to Reviewers_v2.pdf [file pone.0323544.s013.pdf]

Response to Reviewers PONE-D-24-34740

Dear Editor Gordin and Reviewers,

Thank you for managing review of our manuscript **South Arabia's prehistoric monument landscape shows social resilience to climate change,** submitted to PLOS ONE 13 August 2024, manuscript number PONE-D-24-34740. In returning revisions, we have paid close attention to your instructions and to the suggestions of reviewers, for which we are truly grateful. Per your request, we are uploading 1) a marked-up copy that highlights changes ("Revised Manuscript with Track Changes") and 2) an unmarked version of our revised paper ("manuscript").

Below please find a point-by-point response, first to your editor's letter and next, to each reviewer. Line numbers refer to a version with TRACKED CHANGES VISIBLE.

Editor's Letter

#1. We have followed PLOS ONE's style requirements as indicated at the online locations.

#2. You mention that the 'Funding Information' and 'Financial Disclosure' sections do not match. In our resubmission, we tried to use the same statement for both sections, but the submission system still prints the first submission statement. The following is a revised statement for both "Funding Information" and "Financial Disclosure" sections. It has been edited from our original submission and produced here (italics)

This research has been funded by the US National Science Foundation under the Coupled Human - Natural Systems Large Grants (CNH-L 1617185) (PI-JMcC); and the US National Science Foundation Human Social Dynamics Program (DHB 0624268) (PI-JMcC); funds contributing to field work came from a National Geographic Explorer Grant # EC-44704R-18 (PI-AFB) and the Ministry of Heritage and Tourism, Sultanate of Oman (J.McC, A.A.Al-K, A.Al-M). The funders had no role in study design, data collection and analysis, decision to publish, or preparation of the manuscript.

#3. This work was not previously peer reviewed, nor published in whole or in part in the peer-reviewed literature. Our analysis is new, as are the data. Exceptions are the open access oxygen isotopic data in Figure 2 (obtained from Fleitmann et al. 2003, 2007 [54]). Some excavated tombs have been individually documented in a (non-peer reviewed) book we cite (McCorriston 2023) [55] where the overlap is some radiometric dates appearing in Figure 2 and described in Supplemental Table 3. Figure 2 also juxtaposes the published Bayesian posteriors from McCorriston & Dye 2020 [38] with other climate and radiometric data, thereby providing a new paleoclimate timeline in the top register of Figure 2. It is

normal and innovative to synthesize data from multiple sources—some already published—to address a new focus with a new analytical approach and new data, as we have done here. There are many precedents for compiling paleodata from different open sources and researchers to arrive at new analytical conclusions (e.g., Petraglia et al. 2020 [26], Lézine et al. 2010 [24], 2017 [44]), including previous PLOS ONE publications (e.g., Crassard et al. 2020 https://doi.org/10.1371/journal.pone.0236314)

#4. We have included an ethics statement in the 'Methods' section. Our study involved field research (overseas, international). It did NOT include human participants, human specimens or tissue, vertebrate animals or cephalopods, vertebrate embryos or tissues. Our statement reads: (italics) (Lines 213-215)

In conformity with ethical standards, we conducted all field research under (un-numbered) permits issued annually through 5-year agreements signed between Ministry of Heritage and Tourism, Sultanate of Oman and Joy McCorriston (2007-2013, 2017-2021).

#5. All data are included in the manuscript with Supplemental Files and placed (also) in an online data repository (tDAR-the Digital Archaeological Record. Open data access will occur with publication, without restrictions.
For tDAR details, please see "Describe where the data may be found in full sentences..." section of the original submission statement. (We apologize for initial confusion over access at time of submission).

We note that in *Comments to the Author #3*, both reviewers found "**Yes**" to the question, "Have the authors made all data underlying the findings in their manuscript fully available?"

#6. Remove Identifying Data. This is confusing to us. There are no human research participants. Therefore there is no identifying data in the manuscript or Supporting Information files. Is it possible that column headers such as "Code," "Distribution & Date," place names (not people's names) in the data, or the names of classes (of monuments) were erroneously flagged by an AI assistant during manuscript processing?

We have uploaded the data files again after careful consideration of #6.

#7. Maps have been reproduced using CC-BY-4.0 open datasets. Specifically, Mapzen terrain for the elevation raster, and geoBoundaries datasets for country (global ADM0) and region (ADM1) boundary polygons.

#8. We have reviewed all references to ensure they are complete, formatted correctly, and conform to your instructions regarding retractions. Per the reviewers' comments, we added the following references to support clarifications and contextualization of our research:

31. Bocinsky and Kohler 2014

33. Burke et al. 2021

36. Burke, Riel-Salvatore, and Barton 2018 Caramanica et al. 2020 [32]

37. Chirikure et al. 2024

35. Clarke et al. 2016

64. Evans et al. 2014

27. Fedele 2008

54. Fleitman et al. 2003,2007

22. Howey, Palace, and McMichael 2016

23. Hudson et al. 2022

28. Uerpmann and Uerpmann 2008

Reviewer #1 (Review Comments to the Author)

[we address reviewer's bulleted points as reviewer listed them]

"On a minor note..." --we have corrected for a uniform "BP" throughout abstract, text and table 2

- Monument survey methods. The reviewer seems to imply that the survey *methods* presented are not new, and this is indeed the case. For readers who may not have seen our prior publications, including those in a low circulation journal *(J Oman Studies)*, we have 3 paragraphs summarizing how our data were collected and importantly (new), showcasing that the sampling over multiple years and projects nevertheless consistently collected all monument data within a defined viewshed (sampled area). We have edited the text for clarity on these points.
- Chronology. The reviewer advises adding "a more adequate and in-depts discussion of chronology..." We have added a section heading, "Chronological and stratigraphic methods" (Line 283) to discuss the absolute dates, the sample sizes, reuse of monuments, stratigraphic implications of samples, target events and dates. We have also amplified our discussion of the chronology presented here and its context within regional chronology (Hadramawt, Arabian Peninsula). Finally, in this expanded section, we also provide additional discussion of the dating of SCABS. (Lines 296-316)

Radiocarbon determinations come from excavated samples with varying potential to date the initial construction of a monument. Sometimes dated samples are from bone (human and faunal) and determine when an organism died. Other samples are carbon from charcoal, buried humus, or shell. We used stratigraphic associations to contextualize how the deposition of such samples relates to the actual event of monument construction. With multiple monuments of each type, we documented construction materials and techniques, preservation, use, and re-use, including subsequent burials and sacrifices, evidence of collapse, additions, and subsequently quarried stone [16, 55, 71, 72, 73]. The probability determinations in Figure 2 represent events prior or posterior to monument construction. Also, as a responsible reporting of results, we include a radiocarbon determination we think has unexplained error (D033-001). Another determination (D014-002) also lies outside our expected range. (For stratigraphic details see [14, 55]). More samples would surely refine and constrain our chronology, but we caution that excavation is a costly and sometimes unsuccessful path to obtain contextualized radiocarbon samples. Nevertheless, excavation remains the only viable strategy for a classification anchored in absolute dates.

Our classification and chronology largely correlate with observations across Arabia [3, 13, 15, 17, 18, 19, 20, 34, 39, 40, 74]. While multiple researchers have—like us—found episodes of re-use in existing monuments, we focus on the pattern of initial construction our studies of stone placements, arrangements, inclusions, additions, and radiocarbon dates indicate. As we documented through excavation, the reuse of HCT monuments occurred sometimes millennia after construction and disuse. This circumstance extended the HCT timeline in Hadramawt (pink dotted line in Figure 2). Elsewhere in Oman, Williams and Gregoricka [40] document re-visits and deposits subsequent to SCAB construction. Our SCAB sample (n=23) is undated, but we find some that share attributes of Neolithic Platforms, such as a standing stone and East-facing façade (e.g. D106-002); others are only some meters from Neolithic Platforms (e.g.,D027-001, D027-003). (S2-Table, S3-Table).

Finally, we have updated the caption for Figure 2 to clarify the reuse of monuments (evident from stratigraphic excavations)

The long dotted line for HCT reflects stratigraphic evidence of re-use centuries and millennia after primary interment

- Construction techniques. Reviewer requests further explanation for differentiation between "episodic" and "accretive" monuments. We have added manuscript text that describes which variables separate episodic from accretive, and we clarify that the classification refers only to initial construction, not to subsequent visits and modifications. (Lines 222-230, Lines 255-275). We also note that the removal of the file "Description of Monument Types.xlsx" per Editor's Letter #6 (above) may have walled off reviewer's access to this information in our submission.

Our terminology refers to the initial construction of monuments according to a structural-cognitive template. We recognize, as both stratigraphic examples and in theoretical concept, that monuments were re-visited, altered, additions made after substantial lapses in time, sometimes partially or fully dismantled, and appropriated—such is the role of

monuments in both shaping and being shaped by social groups as literal touchstones for institutional thought [41, 67]. For example, through radiocarbon determinations, we documented instances of animal offerings placed into tombs opened and re-sealed a thousand years after a dated burial. This use is unrelated to initial construction, which was the target of our documentation through chronology and measurements.

For those that we could assign, we used the following visual criteria. Platforms have large blocks or upright slabs forming an outer base and an overall D-shaped or oval plan. Often the largest uprights form an eastern face. Platforms have a fill of smaller stones, and typically also have a (sometimes worked) standing stone set further to the east. (Excavations inside show that the uprights were supported by interior chock stones, in turn held in place by fill.) To balance and support stones and consolidate them into a free-standing, stable platform required rapid (i.e., episodic) initial construction. An HCT appears as a conical cairn 3-5 m across with a depressed center. This depression denotes an interior (usually collapsed) chamber made from upright or stacked slabs. Usually some of the chamber facing is still visible without excavation. Tomb (HCT) construction was surely also episodic, given the exigency of burial in a hot environment populated by scavengers and carnivores. A SCAB is also a conical structure, created by stacking boulders in concentric daises decreasing in diameter with the height of the monument. Triliths have clusters of 1-3 upright stones supported by a very low, oblong, gravel platform outlined by cobbles. It would be difficult to mistake this distinctive monument for any other type (Fig 1). Each trilith platform and its adjacent hearths is repeated along an alignment. We identified boat graves by their shape in plan and by the clustering of multiple, abutting, boat-shaped elements (Fig 1), each of which likely included a burial. In the cases of agglomerated triliths and boat graves, there is no construction characteristic that requires completion in a single episode. Triliths and boat graves could and sometimes did appear as isolated, unreplicated elements; this indicates that conglomerated examples were accretive, potentially completed over many years.

- <u>Monument description and sample size</u>. Per reviewer's suggestion, we added a column (N "excavated") in the S1-Table.xlsx (formerly "Monuments Data Table ST-1"). We also included a small paragraph discussing sample size and the inherent constraints on sampling. We have already explained that the regularized multinomial logistic regression "was preferred over machine learning techniques ....as it is more robust to the unequal numbers of observations of different monument types." We note prior studies on the statistical significance of distributions across different landforms, and we suggest that our survey methods account for what has been established about these monuments in prior studies (Lines 702-709)

Large differences exist in the numbers of examples we recorded for each monument type. Prior experience in a comparable landscape (Hadramawt, southern Yemen) demonstrated that these numbers are not significantly explained by preservation conditions of different landforms. Moreover, platforms are significantly associated with lower elevations and HCT in higher elevations [58], both captured in our viewshed boundary in this research so that our monument counts would be relatively unaffected by terrain differences. Therefore other factors—such as period, cultural conventions, available labor—account for differences in the numbers of monuments of each type.

Per reviewer's comment, we have added some broader information on the study area and the subsistence changes (Lines 76-82).

Albeit from few studies, Arabian faunal remains also suggest that cattle herders thrived in wetter environments in the early to mid-Holocene and adjusted their focus to different taxa according to available graze and browse as climates changed [27-29] so that cattle herders preferred wetter environments than today's. In Dhofar's steep environmental gradient, cattle herders today remain in the forested mountains and narrow plateau near the coast [30]. The arid interior is today sparsely populated by mobile goat and camel herders.

- Conclusion. Per reviewer's suggestion, we have formulated and added to a short conclusion: (Lines 823-831)

Derived from the metrics of labor scheduling and location, and testable in new data sets, our empirical model theorizes eras of monument building as the exercises of social capacities to absorb risk in the dynamic climates and environments of the arid sub-tropics of Arabia. By decentering analysis from a single era or type of monument, we show how monument classes are proxies for social behavior of mobile, persistent pastoralists. Our model highlights a reliance on monuments as touchstones of social belonging and as flexible technologies of social resilience in a changing world. While built on archaeological observations of monuments in Arabia, our model may be applicable and adaptable to assess social resilience in other regions, such as Saharan, Mongolian, or the high Andes.

Reviewer #2 (Review Comments to the Author)

- Methodological Details. Both reviewers requested criteria for categorizing monuments into episodic and accretive constructions (addressed per Reviewer 1 above).

The reviewer has asked for simpler explanations of the analytical methods. We have edited these sections, specifically the methods on ML and regularized regression, reduced the use of jargon and removed non-essential technical explanation. We have provided further explanation or examples for unavoidable technical terms. We hope this makes the manuscript more readable and accessible to a range of audiences.

- Environmental and Cultural Context. While we recognize and echo the reviewer's interest in the deeper cultural significance of monuments—how they functioned within social networks of ancient pastoralists—we have declined to include greater detail in this (already long) manuscript. These issues are properly the topics of different methodological and analytical approaches, deserving of fuller treatment than can be offered here. One author (McCorriston) has already explored these through multiple

published articles (McCorriston et al. 2012, McCorriston et al. 2018, both cited in manuscript and references [51, 42]) and several books (McCorriston 2011, McCorriston and Harrower, eds., 2020, now cited in manuscript and references [96, 16, 57, 38, 100, 71, 58]). These cited works consider the cultural significance of Arabian monuments very much along the arguments this reviewer raises (territorial boundaries, social ties, social constitution, pilgrimage rituals). Instead, we include a sentence referencing these publications. (Line 669)

Monuments had culturally specific meanings [51,96].

And Lines 720-721

HCT would have higher prominence on bare terrain*, perhaps consonant with the territorial signaling archaeologists have suggested as their function [8, 102].*

- Statistical Analysis and Visualizations:

Figure 5 is a forest plot. They are one of the most interpretable plot types for presenting model coefficients. Our plot does not include standard error bars, making it even simpler to interpret than conventional forest plots. There is no simpler way to present these results. The caption text provides a very clear explanation of how to interpret the plot. Moreover, two paragraphs, totaling 170 words, guide the reader through a summary of the main takeaways. We have revised the text to explicitly reference the figure at the start of the textual explanation of the plot.

The manuscript states that the statistics were carried out in R, and specifies which R packages were used. Moreover, we cite the methodological papers behind the analytical methods implemented in the R packages. With this information, the reader can easily find further reading material on these methods.

- Theoretical framework. The reviewer makes good points, to which we responded. Throughout we have clarified that among the coping, adaptive, and transformative dimensions of resilience, we see the adjustment of monument technologies as an adaptive resilience (as opposed to settling in oases and initiating agriculture, which we consider transformative and describe as such with the addition: (Lines 677-681—regrettably the "tracked changes" in this passage were "accepted," but it is new)

We consider monuments as assets that facilitated social belonging and were technically adjustable to sustain social cohesion for desert pastoralists who did not abandon herding with environmental changes. Such changes included reduced graze, drying springs, changes in frequency and distributions of seeps, and in some eras, amelioration of rangeland.

And Lines 94-96-- regrettably the "tracked changes" in this passage were "accepted," but it is new

Some strategies manifest truly transformative resilience, such as the settlement of oases and inception of Arabian agricultural systems. Other strategies reflect an adaptive social resilience, manifest in the changing styles of Arabian monuments.

- Collocation Analysis. (Line 710-711) We added:

Results of collocation and environmental analyses suggest that historic place attachments alone cannot explain spatial distributions of different monument types.

And (Lines 732-733) also added to address reviewer's valid comment about results that challenge initial hypothesis.

Our analysis scrutinizes and quantifies these purported relationships, *explaining why monuments of different eras do not appear to cluster as historic attachment to place.*

- Global Relevance. Both reviewers requested information on global relevance and general aspects. While we do not provide a comprehensive review of all research on monumental landscapes in our already lengthy analysis, we have inserted text and references in the introduction that include diverse examples and approaches from South America, East Africa, Saharan Africa, southern Africa, North American Southwest, Europe, Near East, and elsewhere in Arabia (Lines 86-94-- regrettably the "tracked changes" in this passage were "accepted," but it is new)

Our study complements recent studies of archaeological cultures and climate proxy records that consider the impact of aridification phases on prehistoric populations [21, 31-32]. Diverse cultures in diverse circumstances practiced different strategies for resilience [33]. These include changes that are manifest as, for example, changes in settlement and mobility, shifting resources and adopting new ones, engineering flexible field and water catchment systems, adjusting exchange networks, and employing traditional ecological knowledge and cosmology in a changing world [34-37].

Thank you for your further consideration of our manuscript and thanks to reviewers for their patient and careful comments. We hope it now merits publication in PLOS ONE.

Sincerely,

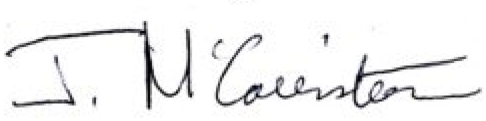

Joy McCorriston (for the authors)
